# Efficacy of Zhuyu Pill Intervention in a Cholestasis Rat Model: Mutual Effects on Fecal Metabolism and Microbial Diversity

**DOI:** 10.3389/fphar.2021.695035

**Published:** 2021-09-02

**Authors:** Han Yu, Chao Liu, Fenghua Zhang, Jianfei Wang, Jun Han, Xin Zhou, Yueqiang Wen, Tao Shen

**Affiliations:** ^1^ School of Basic Medicine, Chengdu University of Traditional Chinese Medicine, Chengdu, China; ^2^ Department of Nephrology, South of Guang’anmen Hospital, Beijing, China; ^3^ Department of Reader Service and Culture Education, Chengdu University of Traditional Chinese Medicine, Chengdu, China

**Keywords:** cholestasis, zhuyu pill, fecal metabolism, fecal microbial diversity, mutual effects

## Abstract

Cholestasis is a clinical condition resulting from impaired bile flow. Currently, patients with cholestasis face several barriers in seeking diagnosis and treatment. Zhuyu Pill (ZYP) is an ancient classic formula of the Coptis-Evodia herb couples (CEHC), and has been used for cholestasis treatment in the clinic, however, its underlying biological activity in cholestasis remain to be clarified. In this study, an α-naphthyl-isothiocyanate (ANIT, 50 mg/kg)-induced rat model of cholestasis was treated with ZYP. Serum biochemical indices and histopathological evaluation was performed, together with the metabolomics analyses of feces and 16S rDNA sequencing of the fecal microbiota. We evaluated the anti-cholestatic activity of ZYP and investigated the mechanisms underlying its correlation with fecal microbiota and fecal metabolite regulation. The relationships between biochemical indices and changes in gene expression associated with liver injury, levels fecal metabolites, and composition of fecal microbiota were analyzed. The results showed that both high (1.2 g/kg) and low (0.6 g/kg) doses of ZYP could effectively improve biochemical parameters in the blood of cholestasis-induced rat models; the intervention effect of high dose ZYP was superior to that that of lower dose ZYP. Based on a metabolomics test of fecal samples, significantly altered metabolites in the ANIT and ZYP treatment group were identified. In total, 734 metabolites were differentially expressed, and whose biological functions were mainly associated with amino acid metabolism, steroid hormone biosynthesis, and bile secretion. In addition, sequencing of the 16S rDNA unit in fecal samples revealed that the ZYP could improve the fecal microbiota dysbiosis that ANIT had induced. Therefore, we conclude that ANIT altering of blood biochemical and metabolic profiles and of fecal microbiota could effectively be alleviated with ZYP treatment. This study contributes to the “TCM wisdom” applied in clinical diagnosis and treatment of cholestasis.

## Introduction

In recent years, changes in dietary habits and living environments, the incidence of liver diseases, and associated complications represent a major healthcare burden in China (Tang et al., 2017). Cholestasis, which can be caused by pre-existing medical conditions including infections, drug treatment, and metabolic or genetic disorders (Boyer, 2007), is classified as intrahepatic, involving mainly liver parenchymal cells, or extrahepatic, involving any excretory block outside of the liver (Chatterjee et al., 2020). A cross-sectional study of cholestasis in 1000 patients in China demonstrated that, the biochemical parameters in the blood of intrahepatic cholestasis, such as alkaline phosphatase (ALP) and γ-glutamyl transferase (γ -GT), were higher than the upper limit of normal, and the risk and severity of liver damage in these patients were markedly increased (Cheng et al., 2015). Due to the lack of timely treatment, liver cells, including portal myofibroblasts and hepatic stellate cells, are hyperactivated and cholestasis leads to fibrosis and even cirrhosis (Allen et al., 2011). Currently available therapeutic options of cholestasis are limited.

Bile acids such as ursodeoxycholic acid (UDCA) and chenodeoxycholic acid (CDCA) are used routinely for the dissolution of gallstones and have recently been proposed as treatment for chronic cholestatic liver disease (Crosignani et al., 1996; Beuers et al., 1998; Sasaki et al., 2001). However, approximately one-third of patients achieve little or no response to UDCA treatment (Itakura et al., 2004; Lane and Murray, 2017). Traditional Chinese medicine (TCM) has been used to treat liver and digestive disease in China since ancient times. *Coptidis rhizoma* (Coptis, Huanglian in Chinese) and *Evodiae fructus* (Evodia, Wuzhuyu in Chinese) is a well-known herbal combination usually used to treat liver and digestive diseases. *Zuojin Pill* (ZJP, usually in a mixture ratio 6:1, g/g) and *Zhuyu Pill* (ZYP, usually in mixture ratio 1:1, g/g) are the ancient classic formulas of the Coptis-Evodia herb couple (CEHC). To date, studies have shown that ZJP can influence the metabolism of venlafaxine (Cheng et al., 2011), the brain-gut axis, and cell proliferation, and its therapeutic effects in liver and stomach diseases have been reported (Wang et al., 2015; Guo et al., 2019). These results support the CEHC combination for the treatment of liver and digestive diseases. However, compared with ZJP, less attention has been paid to ZYP and the efficacy and effector mechanisms of ZYP remain to be examined.

TCM is a complex therapy involving multiple targets with synergistic or antagonistic interactions among its components. In contrast to the Western medicine notion of “one target, one drug,” TCM is characterized by “multi-component, multi-target, and multi-pathway” activity; this concept of the holistic view is emphasized in the theory of TCM. Metabonomics is an important technique in systems biology (Kaddurah-Daouk et al., 2008). It is the study of the composition and variation of metabolic groups, and thereby reveals the overall metabolic response and dynamic changes induced under different conditions. Metabonomics has been used to study the organism as a whole, which is consistent with the holistic view of TCM and the concept of syndrome differentiation and treatment (Wang et al., 2017). Because the modernization of TCM is becoming necessary and urgent (Li et al., 2008), metabolomics represents a modern technology with a great potential toward understanding the efficacy and mechanism of TCM (Sun et al., 2012; Wang et al., 2020).

Intestinal microecology not only comprises the gut microbiota, but also numerous gut microbiota metabolites (Chen C. et al., 2019). Hence, the gut microbiota can be defined as a “metabolic organ” (Wei et al., 2020). Recent studies have shown that, bile acids (BAs) are host-derived and microbial-modified metabolites that regulate both the gut microbiome and host metabolism (Islam et al., 2011; Wahlstrom et al., 2016; Kemis et al., 2019). The fecal metabolome provides unique information on the metabolic interactions between the fecal microbiota, diet, and host. Combined with 16S next-generation-sequencing, the metabolome can provide a comprehensive phenotype of the host-microbiome interplay (Yang et al., 2019).

In this study, an animal model of cholestasis was induced using α-naphthylisothiocyanate (ANIT) (Hill et al., 1999) and the pharmacodynamic effects were evaluated by changes in serum biochemical indices, liver histopathology, liver inflammation, apoptosis, and gene expression associated with fibrosis. Differential metabolites levels were identified using liquid chromatography-mass spectrometry (LC-MS) with an untargeted metabolomics approach and the abundance of fecal microbiota was analyzed using 16S ribosomal RNA (16S rRNA). In addition, to characterizing the mechanisms of ZYP activity in regulating cholestasis, we aimed: *1*) to explore the effects of ZYP on improving liver function; *2*) to measure the composition of the fecal microbiota in order to identify differential metabolites induced of ZYP in cholestasis; and *3*) to correlate the results of untargeted metabolomics and the diversity of fecal microbes to better define the underlying biological mechanisms involved in the anticholestatic activity of ZYP. These findings will enhance our understanding of pathogenic mechanisms of cholestasis and will provide a new therapeutic option for the treatment of cholestasis.

## Materials and Methods

### Reagents

ANIT was purchased from Sigma-Aldrich Co. (St. Louis, MO, United States). *Coptidis rhizoma* and *Evodiae fructus* were purchased from Beijing Tongrentang Science and Technology Development Co. Ltd (Beijing, China). Water, methanol, acetonitrile, and formic acid were purchased from the CNW Technologies GmbH (Düsseldorf, Germany). L,-2-chlorophenylalanine was from Shanghai Hengchuang Biotechnology Co., Ltd. (Shanghai, China). All chemicals and solvents were analytical or HPLC grade.

### Preparation and Quality Control of ZYP

Zhuyu Pill was prepared as follows: *Coptidis rhizoma* (6 g), and *Evodiae fructus* (6 g) were weighed and placed in 120 ml of distilled water (1:10, w/v) for 6 h. These samples were then boiled twice, for 45 min each time.

To determine the active constituents, two main alkaloids (berberine and coptisine) in ZYP were analyzed by HPLC using the Agilent 1260 Infinity II (Agilent Technologies Inc., California, United States). The chromatographic separation was carried out with a Welch Ultimate XB-C18 Column (4.6 mm × 250 mm, 5 µm, Maryland, United States) at a column temperature of 30°C. The linear-gradient mobile phase consisted of mobile phase A (50 mM monopotassium phosphate+0.4% sodium heptane sulfonate, pH = 4) and mobile phase B (pure methanol). The gradient of the mobile phase was utilized (0–15 min, 95% A, 5% B; 15–40 min, 50% A, 50% B; 40–55 min, 30% A, 70% B, 55–60 min, 95% A, 5% B), and a flow rate of 1.0 ml/min was adopted. The detection wavelength was set as (0–44 min, 345 nm; 44–48 min, 226 nm; 48–60 min, 345 nm). The HPLC analysis showed that the contents of berberine and coptisine in ZYP were 33.2 and 13.4 mg/g, respectively.

### Animals and Treatments

A total of 24 male Sprague-Dawley rats (7–8 wk old; weighing 260 ± 20 g) were obtained from Beijing HFK Bioscience Co., Ltd. (Beijing, China; certification no. SCXK-JING 2019-0008). All animals were allowed to acclimate for 1 wk prior to the experiments and were maintained at a constant temperature (25 ± 2°C) and 50% humidity with a 12-h/12-h light/dark cycle; all the rats had access to water and food *ad libitum*.

An overview of the experimental design is presented in Table 1. The animals were randomly divided into four groups of six rats each, including the control, model, ZYP-low dose (ZYP−) and ZYP-high dose (ZYP+) groups. The rats in the normal group served as the normal control and were given distilled water each day and treated with vehicle (olive oil) alone. The model group was treated with 50 mg/kg ANIT dissolved in an equal volume of olive oil by gavage (Chen et al., 2016). ZYP was given to the treatment groups at doses of 0.6 (ZYP−), 1.2 (ZYP+) g/kg body weight, respectively, six times before and four times after they were treated with 50 mg/kg ANIT by gavage. In this study, ZYP doses that were adopted were based on the maximum recommended clinical dose (MRCD, 12 g/60 kg/day).

**TABLE 1:** The schematic diagram of the animal experimental design.

Group	week old	1–7 days	8–13 days	14 days	15–18 days	19 days
Control	7–8	acclimate	distilled water	Olive Oil	distilled water	Sacrifice
Model	7–8	acclimate	distilled water	50 mg/kg ANIT	distilled water	Sacrifice
ZYP−	7–8	acclimate	0.6 g/kg ZYP	50 mg/kg ANIT	0.6 g/kg ZYP	Sacrifice
ZYP+	7–8	acclimate	1.2 g/kg ZYP	50 mg/kg ANIT	1.2 g/kg ZYP	Sacrifice

## Ethics Approval and Consent to Participate

The study protocol was performed in strict accordance with the recommendations of the Guidelines for the Care and Use of Laboratory Animals of the Ministry of Science and Technology of China, and was approved by Ethics Committee of Chengdu University of Traditional Chinese Medicine (Chengdu, China). All efforts were made to minimize animal suffering.

### Sample Collection, Liver Function, and Gene Expression Assays

The rats were provided with standard chow and water on completion of treatments. At least two fresh stools pellets were obtained from each rat after the last ZYP administration. Samples were placed in sterile conical tubes and immediately frozen at −80°C until further metabolic profiling and microbial community analysis, respectively.

According to pilot experiments, the rats were then fasted for 12 h, and were then euthanized 12 h after the last ZYP administration. Blood samples were collected from the inferior vena cava and the liver was excised from each rat immediately after sacrifice. The blood samples were collected and centrifuged at 3500 × g and 4°C for 15 min to obtain the serum. The serum total cholesterol (TC; cat. no. 105-000448-00), aspartate aminotransferase (AST; cat. no. 105-000443-00), alanine aminotransferase (ALT; cat. no. 105-000442-00), γ-glutamyl transpeptidase (γ-GT; cat. no. 105-000445-00), total bilirubin (TBIL; cat. no. 0041-30-53548) and total bile acid (TBA; cat. no. 105-000456-00) levels were detected using a fully automatic biochemical analyzer (BS-240VET), which together with all assay kits were purchased from Mindray Bio-medical Electronics Company (Mindray Bio-medical Electronics Company, Ltd, Shenzhen, China).

Total RNA of liver samples was extracted by mirVana™ RNA Isolation Kit (Cat. AM1561; Thermo Fisher Scientific, Waltham, MA, United States). The expression of tumour necrosis factor alpha (TNF-α), transforming growth factor beta 1 (TGF-β1), interleukin-10 (IL-10), metalloproteinase inhibitor 1 (TIMP-1), Glutamate cysteine ligase catalytic subunit (GCLC), Glutamate cysteine ligase regulatory subunit (GCLM), B-cell lymphoma 2 (BCL2) and BCL2 associated X (BAX) were detected by Quantitative Real-time PCR (qPCR). Quantification was performed using a two-step reaction process: reverse transcription (RT) and PCR. Each RT reaction (10 μl total) consisted of 0.5 μg of RNA, 2 μl of 5×TransScript All-in-one SuperMix for qPCR, and 0.5 μl of gDNA Remover. Reactions were performed in a GeneAmp® PCR System 9700 (Thermo Fisher Scientific) for 15 min at 42°C and then 5 s at 85°C. The RT reaction mix was then diluted 10× in nuclease-free water and held at −20°C.

Real-time PCR was performed using a LightCycler® 480 Ⅱ Real-time PCR Instrument (Roche, Basel, Switzerland) with a 10 μl PCR reaction mixture that included 1 μl of cDNA, 5 μl of 2×PerfectStartTM Green qPCR SuperMix, 0.2 μl each of forward and reverse primers, and 3.6 μl of nuclease-free water. Reactions were incubated in a 384-well optical plate (Roche, Basel, Switzerland) at 94°C for 30 s, followed by 45 cycles of 94°C for 5 s and 60°C for 30 s. Each sample was analysed in triplicates. At the end of the PCR cycles, melting curve analysis was performed to validate the specific generation of the expected PCR product. The primer sequences were designed in the laboratory and synthesised by TsingKe Biotech based on the mRNA sequences obtained from the NCBI database (Table 2).

**TABLE 2:** mRNA primer sequences.

Gene Symbol	Forward primer (5->3)	Reverse primer (5->3)	Product length (bp)	Tm (°C)
TNF-α	5ʹ-CCC​TGG​TAT​GAG​CCC​ATG​TA-3ʹ	5ʹ-CGG​ACT​CCG​TGA​TGT​CTA​AGT​A-3ʹ	106	60
TGF-β1	5ʹ-TAC​GTC​AGA​CAT​TCG​GGA​AG-3ʹ	5ʹ-TAC​GTG​TTG​CTC​CAC​AGT​T-3ʹ	97	60
IL-10	5ʹ-CTT​TAA​GGG​TTA​CTT​GGG​TTG​C-3ʹ	5ʹ-TTT​CTG​GGC​CAT​GGT​TCT​C-3ʹ	95	60
TIMP-1	5ʹ-GAT​ATG​TCC​ACA​AGT​CCC​AGA-3ʹ	5ʹ-CAG​TGA​TGT​GCA​AAT​TTC​CG-3ʹ	81	60
GCLC	5ʹ-CCC​TCT​TCT​TTC​CAG​ACG-3ʹ	5ʹ-TGG​CAC​ATT​GAT​GAC​AAC​C-3ʹ	104	60
GCLM	5ʹ-GTG​TGA​TGC​CAC​CAG​ATT-3ʹ	5ʹ-TTG​CCT​CAG​AGA​GCA​GTT​C-3ʹ	93	60
BCL2	5ʹ-GAT​TGT​GGC​CTT​CTT​TGA​GT-3ʹ	5ʹ-CAC​AGA​GCG​ATG​TTG​TCC-3ʹ	90	60
BAX	5ʹ-TGA​GCT​GAC​CTT​GGA​GCA-3ʹ	5ʹ-GTC​CAG​TTC​ATC​GCC​AAT-3ʹ	85	60
ACTB	5ʹ-GCG​AGT​ACA​ACC​TTC​TTG​C-3ʹ	5ʹ-TAT​CGT​CAT​CCA​TGG​CGA​AC-3ʹ	72	60

### Histological Analysis of Liver Damage

Liver tissues were excised and fixed in 10% phosphate-buffered formalin. Fixed issues were cut into 1 × 1 × 0.3 cm sections. Sections were dehydrated in a gradient alcohol series, and embedded in paraffin wax blocks. The embedded wax blocks were cut into 4–5 μm thick slices. Following dewaxing slides in xylene, the slides were stained with hematoxylin and agitated for 30 s, rinsed in H_2_O for 1 min, followed by staining with 1% eosin Y solution for 30 s with agitation, all at room temperature (20–25°C). Slides were examined under an Eclipse E100 microscope (NIKON, Tokyo, Japan). The degree of inflammation and necrosis were classified according to the Ishak Scoring System (Ishak et al., 1995; Goodman, 2007).

### Sample Preparation for Metabolome Profiling

A 60 mg stool sample was accurately weighed and transferred to a 1.5-ml Eppendorf tube. Two small steel balls were added to the tube. A 20 μl volume of internal standard (2-chloro-l-phenylalanine in methanol, 0.3 mg/ml) and 600 μl extraction solvent with methanol/water (4/1, v/v) were added to each sample. Samples were stored at −20°C for 5 min and then ground at 60 Hz for 2 min, ultrasonicated at ambient temperature (25–28°C) for 10 min, stored at −20°C for 30 min. The extract was centrifuged at 9800 × g, 4°C for 10 min and then, a 300 μl sample of the supernatant in a brown and glass vial was dried in a freeze concentration centrifugal dryer. Next, 400 μl of a mixture of methanol and water (1/4, v/v) were added to each sample, and samples were vortexed for 30 s, ultrasonicated for 3 min, and then placed at −20°C for 2 h. Finally, samples were centrifuged at 9800 × g, at 4°C for 10 min and the supernatants (150 μl) from each tube were collected using crystal syringes, filtered through 0.22 μm microfilters, and transferred to LC vials. The vials were stored at −80°C until LC-MS analysis. QC samples were prepared by mixing aliquots of the all samples to form a pooled sample. All extraction reagents were precooled at −20°C before use.

### LC-MS Analysis

A Dionex Ultimate 3000 RS UHPLC system fitted with Q-Exactive quadrupole-Orbitrap mass spectrometer equipped with heated electrospray ionization (ESI) source (Thermo Fisher Scientific) was used to analyze the metabolic profiles in both the ESI positive and ESI negative ion modes. An ACQUITY UPLC HSS T3 (100 mm × 2.1 mm, 1.8 μm) was employed in both positive and negative modes. The binary gradient elution system consisted of (A) water (containing 0.1% formic acid, v/v) and (B) acetonitrile (containing 0.1% formic acid, v/v) and separation was achieved using the following gradient: 5–20% B over 0–2 min, 20–60% B over 2–4 min, 60–100% B over 4–11 min, the composition was held at 100% B for 2 min, then 13–13.5 min, 100–5% B, and 13.5–14.5 min holding at 5% B. The flow rate was 0.35 ml/min and column temperature was 45°C. All the samples were kept at 4°C during the analysis. The injection volume was 2 μl.

The mass range was from m/z 100 to 1000. The resolution was set at 70,000 for the full MS scans and 17,500 for MS/MS scans. The collision energy was set at 10, 20, and 40 eV. The mass spectrometer operated as follows: spray voltage, 3800 V (+) and 3000 V (−); sheath gas flow rate, 35 arbitrary units; Aux gas flow rate, eight arbitrary units; capillary temperature, 320°C.

The QCs were injected at regular intervals (every four samples) throughout the analytical run to provide a set of data from which repeatability could be assessed. Raw sequence data of metabolomics have been uploaded to Metabolights (https://www.ebi.ac.uk/metabolights/) and are available through accession number (MTBLS2721).

### Data Preprocessing and Statistical Analysis

The acquired LC-MS raw data were analyzed using the Progenesis QI software (version 2.3; Nonlinear Dynamics, Newcastle, United Kingdom), based on a self-built databases (the EMDB database, is specific for human and animals species, and was set up as an exclusive metabolite database containing data for over 3600 metabolites, including amino acids, lipids, nucleotides, carbohydrates, vitamins and cofactors, and hormones. It also includes information on metabolite structure and mass spectrum data to address metabonomics biology). The following parameters were used: precursor tolerance was set 5 ppm, product tolerance was set 10 ppm, and retention time (RT) tolerance was set 0.02 min. Internal standard detection parameters were deselected for peak RT alignment, isotopic peaks were excluded for analysis, and the noise elimination level was set at 10.00, the minimum intensity was set to 15% of the base peak intensity. An Excel file was obtained containing three-dimensional data sets including m/z, peak RT, and peak intensities, and RT–m/z pairs were used as the identifier for each ion. The resulting matrix was further reduced by removing any peaks with missing value (ion intensity = 0) in more than 50% of the samples. Positive and negative data were combined data, which was then imported into R ropls package.

Principle component analysis (PCA) and Orthogonal partial least-squares-discriminant analyses (OPLS-DA) were carried out to visualize the metabolic alterations between experimental groups, after mean centering (Ctr) and Pareto variance (Par) scaling, respectively. The Hotelling’s T2 region, shown as an ellipse in the score plots of the models, defined the 95% confidence interval (CI) of the modeled variation. Variable importance in the projection (VIP) ranked the overall contribution of each variable to the OPLS-DA model, and variables with VIP >1 were considered relevant for group discrimination. Default seven-round cross-validation and the 200 response permutation test were applied, with one-seventh of the samples excluded from the mathematical model in each round to avoid overfitting.

The differential metabolites were selected based on the combination of a statistically significant threshold of variable influence on projection (VIP) values obtained from the OPLS-DA model and *p*-values derived from a two-tailed Student’s t test using the normalized peak areas, where metabolites with VIP > 1.0 and *p* < 0.05 were considered as differential metabolites.

### DNA Extraction, Library Construction, and Sequencing

Total genomic DNA was extracted using DNA Extraction Kit following the manufacturer’s instructions (Cat.12888; QIAGEN, Dusseldorf, Germany). The DNA concentration was verified with using the NanoDrop and by agarose gel electrophoresis. The DNA genome was used as template for PCR amplification with barcoded primers and Tks Gflex DNA Polymerase (R060B; TaKaRa Bio, Beijing, China). For bacterial diversity analysis, the V3–V4 variable region of the 16S rRNA gene was amplified using primers 343F and 798R (Nossa et al., 2010), using a commercial PCR kit (Cat. 51531; Qiagen).

Amplicon quality was visualized by gel electrophoresis, and amplicons were purified with AMPure XP beads (Cat. A63880; Agencourt), and amplified for another round of PCR. After a second purification with the AMPure XP beads, the final amplicon was quantified using Qubit dsDNA assay kit (Cat. Q32854; Thermo Fisher Scientific). Equal amounts of purified amplicon were pooled for subsequent sequencing based on Novaseq PE250. Raw sequence data were uploaded to the National Center for Biotechnology Information (NCBI) database (https://www.ncbi.nlm.nih.gov/) and are available through accession number (PRJNA720504).

### Taxonomical Annotation

Raw sequencing data were exported in FASTQ format. Paired-end reads were then preprocessed using Trimmomatic software (Bolger et al., 2014) to detect and eliminate ambiguous bases (N). Further, low quality sequences were eliminated having an average quality score below 20 using sliding window trimming approach. After trimming, paired-end reads were assembled using FLASH software (version 34.0.0.118) (Reyon et al., 2012). Parameters of assembly were: 10 bp of minimal overlapping, 200 bp of maximum overlapping and a 20% maximum mismatch rate. Sequences were further filtered as follows: reads with ambiguous, homologous sequences or below 200 bp were abandoned; reads with 75% of bases above Q20 were retained; and, reads with chimera were detected and removed. These steps were achieved using QIIME software (version 1.8.0) (Caporaso et al., 2010b).

Clean reads were subjected to primer sequences removal and clustering to generate operational taxonomic units (OTUs) using Vsearch software (Version 2.4.2) (Rognes et al., 2016) with a 97% similarity cutoff. The representative read of each operational taxonomic unit (OTU) was selected using the QIIME package. All representative reads were annotated and blasted against Unite database (ITSs rDNA) using pynast (v0.1) (Caporaso et al., 2010a).

### Statistical Analysis

Statistical analysis of serum biochemical indexes and gene expression was processed using Graphpad Prism, version v8 (GraphPad Software, Inc., San Diego, United States). All experiments were repeated at least six times and the obtained data are presented as the mean ± standard deviation. The Student’s t-test was used for the analysis of statistical significance between two groups, and one-way analysis of variance followed by Tukey test was applied to analyze statistical significance across three groups or more. A *p-*value < 0.05 was considered to indicate a statistically significant difference.

## Results

### Therapeutic Effects of ZYP on Serum Biochemistry, Liver Inflammation Markers, Cell Necrosis, Fibrosis Mediators, and Liver Histopathology

**FIGURE 1:**
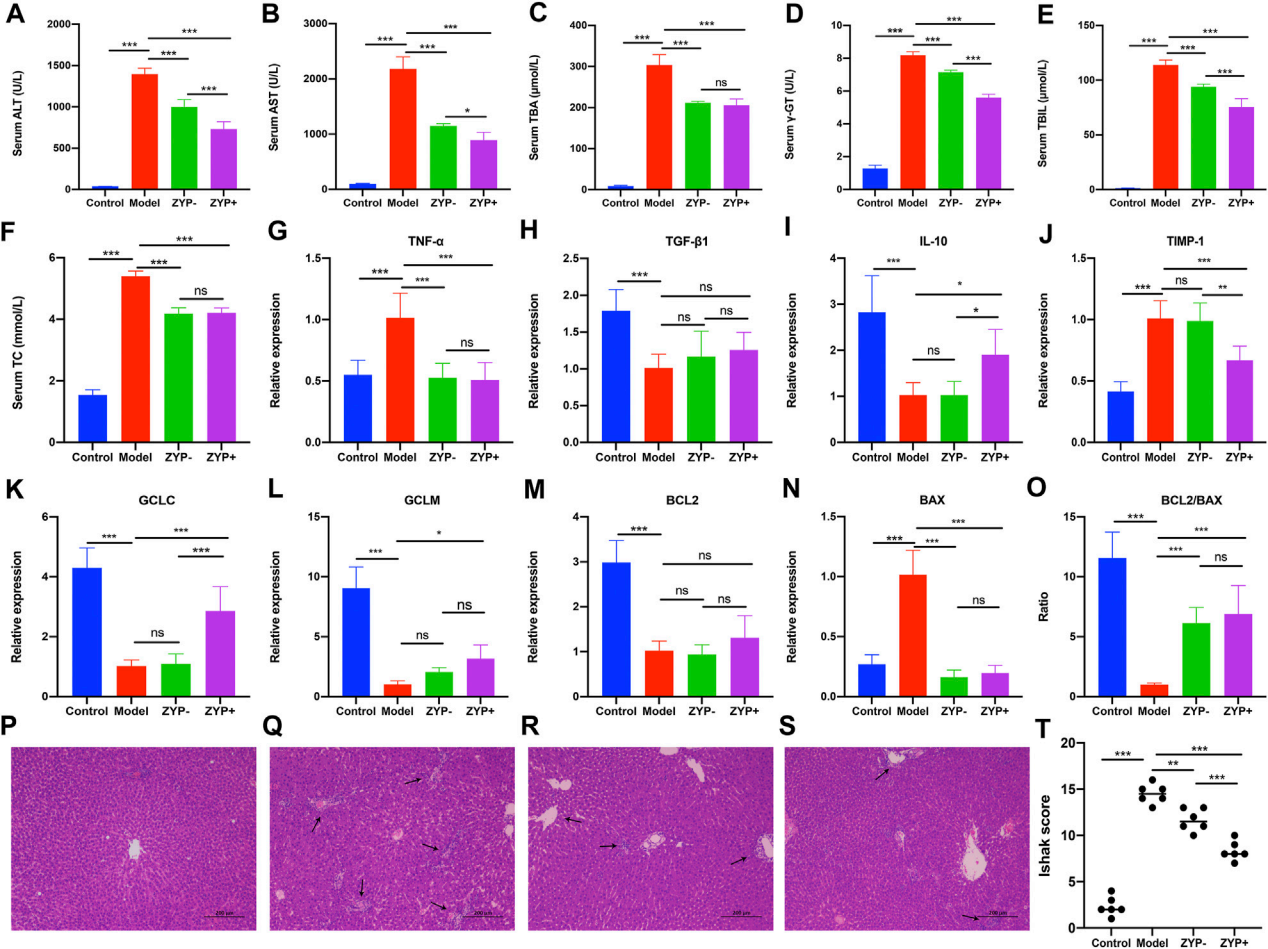
Serum biochemistry, gene expression changes, and histopathological examination of livers evaluating liver injury in Control, Model, ZYP− and ZYP+ groups. **(A)** ALT; **(B)** γ-GT; **(C)** TC; **(D)** AST; **(E)** TBA; **(F)** TBIL; **(G)** TNF-α; **(H)** TGF-β1; **(I)** IL-10; **(J)** TIMP-1; **(K)** GCLC; **(L)** GCLM; **(M)** BCL2; **(N)** BAX; **(O)** BCL2/BAX. Histopathological examination of liver by HE staining at magnification ×100. **(P)** Control; **(Q)** Cholestasis Model; **(R)** ZYP−; **(S)** ZYP+. Arrows indicate the pathological loci. **(T)** Ishak score of hepatic inflammation and necrosis. *n* = 6, ***/**/*, *p* < 0.001/*p* < 0.01/*p* < 0.05; ns, no significant.

As shown in Figure 1, the cholestasis-induced rats exhibited a significant increase in serum ALT and AST levels, which were markedly reduced following treatment with high or low doses of ZYP. Similarly, the levels of TC, TBA, γ-GT, TBIL, the expression of TNF-α, TIMP-1, and the BAX/BCL-2 ratio were significantly increased in cholestasis-induced rats compared with the control rats, and these changes were effectively alleviated following treatment with ZYP. In contrast, the expression of IL-10, GCLC and GCLM showed opposite results, while TGF-β1 showed no significant difference after ZYP intervention (Figures 1A–O). Representative photomicrographs of hematoxylin and eosin (HE)-stained liver tissue from control rats and the cholestasis rats with or without ZYP (0.6/1.2 g/kg) treatment are presented in Figures 1P–S. The Ishak scores are presented in Figure 1T. The morphology of liver lobules was normal in the control group, and the central veins and radiating hepatic cords were clearly visible. In contrast, rats with cholestasis exhibited significantly elevated inflammation and necrosis scores and significant changes in liver structure, including acute infiltration of polymorphonuclear neutrophils, nuclear deformation, and necrosis-induced inflammation. However, the cholestasis-induced rats treated with ZYP, especially the ZYP+ group, presented significantly reduced Ishak scores and exhibited only mild inflammatory infiltration of the central vein and portal area, and relatively milder bile duct epithelial damage.

### Multivariate Statistical Analysis of LC-MS

**FIGURE 2:**
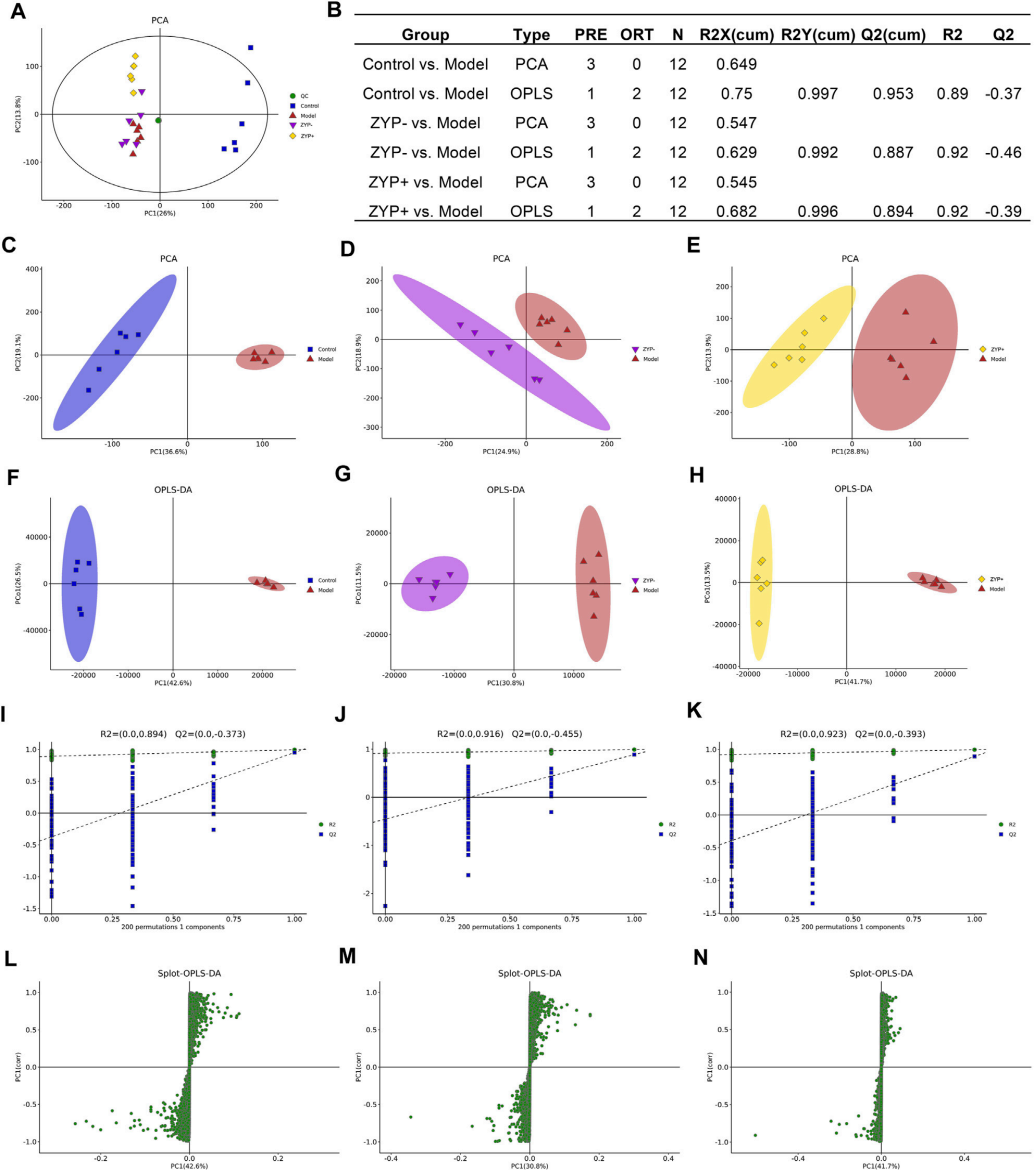
Multivariate statistical analysis of metabolic profiles derived from the Control, Model, ZYP−, and ZYP+ groups. **(A)** PCA score plot of the Control, Model (ANIT), ZYP−, ZYP+ and QC groups; **(B)** Parameters of PCA; **(C**–**E)** PCA of Model vs Control group, ZYP− vs Model and ZYP+ vs Model, respectively; **(F**–**H)** OPLS-DA of the Model vs Control group, ZYP− vs Model and ZYP+ vs Model, respectively; **(I**–**K)** 200-permutation test of the Model vs Control group, ZYP− vs Model and ZYP+ vs Model, respectively; **(L**–**N)** OPLS-DA score plot of the Model vs Control group, ZYP− vs Model and ZYP+ vs Model, respectively.

PCA was used to compare the differences among all groups. As shown in Figure 2, a score plot allowed visualization of the observational clusters, which differed significantly between the control, model, ZYP−, ZYP+, and QC groups (Figures 2A,C–E). The results of the PCA indicated that multivariate statistical analysis was necessary to clarify the differences among the groups; therefore, OPLS-DA models were built to facilitate data interpretation.

OPLS-DA analysis was performed to obtain a deeper understanding of the differential metabolites, which were accountable for the intergroup separation. In this study, the control, model, ZYP−, and ZYP+ groups could be distinguished. The R^2^X, R^2^Y, and Q^2^ (cum) of the model and control groups were 0.75, 0.997, and 0.953 respectively, as compared with the values of 0.682, 0.996, 0.894 (ZYP− vs control), and 0.684, 0.993, 0.866 (ZYP+ vs model), respectively, of the ZYP−/ZYP+ and model groups (Figures 2F–H). To prevent overfitting of the model, the quality of the model was examined by seven-cycle interactive verification and 200-response ranking test. These results indicated that these OPLS-DA were not overfit and indicated that the OPLS-DA achieved high separating capacity (Figures 2I–K). The OPLS-DA loading scatter plot identified several critical variables that were distant from the center of the loading plot, suggesting that these critical variables were important variables for clustering (Figures 2L–N).

### Metabolite Identification and Pathway Analysis in ZYP-treated Rats With Cholestasis

Differential metabolite levels were determined based on VIP values  > 1 and adjusted *p*-values < 0.05. We identified a total of 1090 metabolites that were significantly differentially expressed between the Model and Control groups, and 965 and 734 significantly differentially expressed metabolites between ZYP− and Model group, and the ZYP+ and Model groups, respectively (Supplementary Data).

**FIGURE 3:**
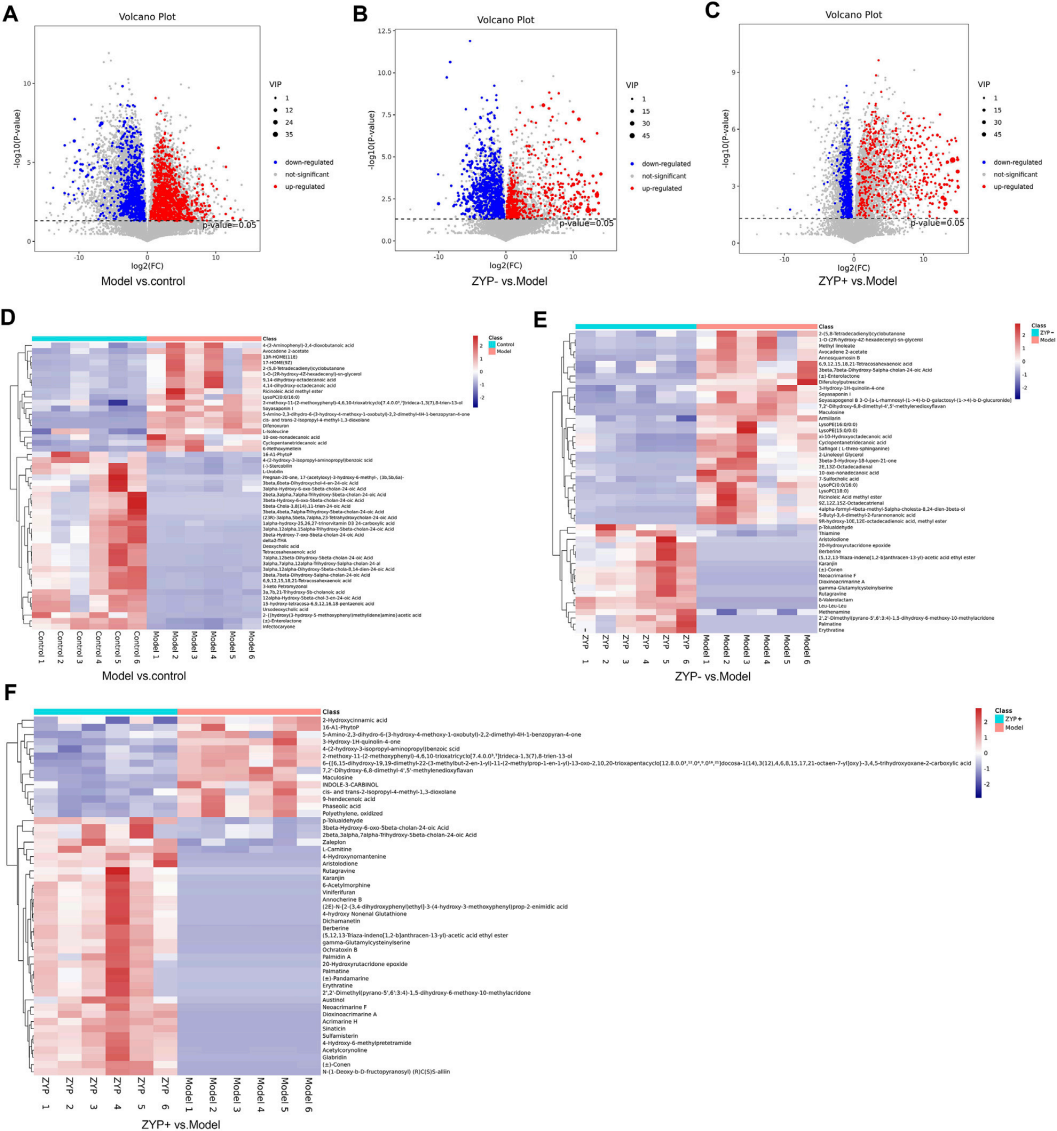
Volcano plot and Heat map of the differential metabolites. **(A**–**C)** Volcano plot of Model vs Control groups, ZYP− vs Model and ZYP+ vs Model groups, respectively; **(D**–**F)** Heat map of Model vs Control groups, ZYP− vs Model and ZYP+ vs Model groups, respectively. Red, blue, and gray represent the upregulated, downregulated, and non-significant changes, respectively.

Based on the results of the serum biochemistry and liver histological examination, the ZYP+ group (1.2 g/kg) exhibited better effects than the ZYP− group (0.6 g/kg). Additionally, the dose of the ZYP+ group is that commonly used in clinical practice; thus for the reasons described above, it is worthy to focus specifically on this group. In the ZYP+ vs Model comparison, the subclasses of differential metabolites analysis was associated with amino acids, peptides, and analogues (54 differential metabolites), fatty acids, and conjugates (37 differential metabolites), and flavonoids (37 differential metabolites). The number and trend of metabolites in different groups were presented in a volcano plot (Figures 3A–C), and the Top 50 differential metabolites between the Control, Model, ZYP+, and ZYP− groups were presented as a heatmap (Figures 3D–F).

**FIGURE 4:**
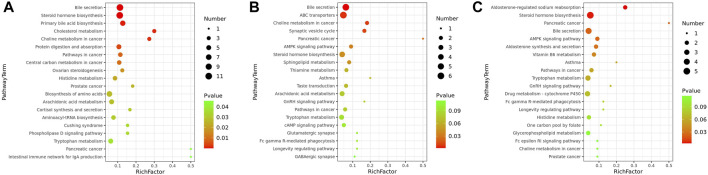
Bubble diagrams of the control, cholestasis model, and ZYP treatment groups. **(A)** Model vs Control group; **(B)** ZYP− vs Model; **(C)** ZYP+ vs Model, respectively. The x-axis shows the Rich factor, the color of each circle indicates the *p*-value, and the size of each circle reflects the number of metabolites of each pathway.

To determine the functions of the differential metabolites, pathway analysis was carried out to further identify potentially enriched pathways (*p* < 0.05) associated with model alterations or ZYP treatment intervention. The results of the significant pathway terms emerging in the Kyoto Encyclopedia of Genes and Genomes (KEGG) analysis were mapped onto a bubble graph (Figure 4). Activation of eight pathways was identified, specifically bile secretion, steroid hormone biosynthesis, choline metabolism in cancer, pathways in cancer, histidine metabolism, prostate cancer, pancreatic cancer, and tryptophan metabolism when compared to the Model vs Control groups and ZYP+ vs Model groups. The above results suggested that ZYP participated in above biological process and signaling pathways and generated significant beneficial effects as intervention of cholestasis.

### Fecal Microbiota Analysis

To explore whether the effects of the ZYP extract were associated with changes in the fecal microbiota, we analyzed the fecal flora composition of rats after ANIT-induced cholestasis or ZYP treatment.

**FIGURE 5:**
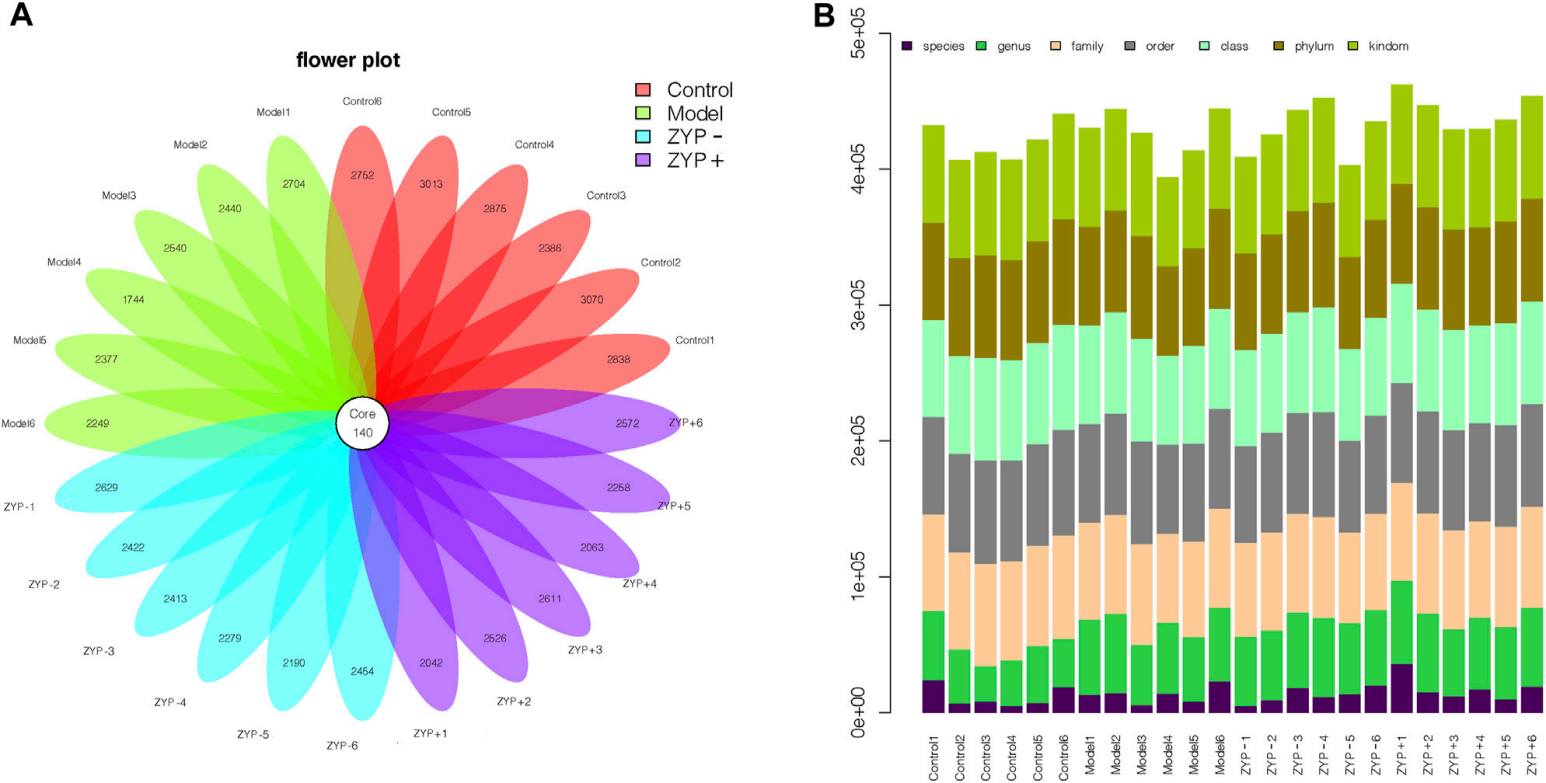
OTUs classification results. **(A)** Operational Taxonomic Units (OTUs) based petal maps; **(B)** Comment situation of tags.

The OTUs at the 97% similarity level were obtained using cluster tags, as is shown in the OTUs-Venn diagram (Figure 5A). In total, 140 OTUs were shared by the Control, Model, ZYP− and ZYP+ groups, the information regarding species, genus, family, order, class, phylum and kingdom level for each sample is shown in Figure 5B.

**FIGURE 6:**
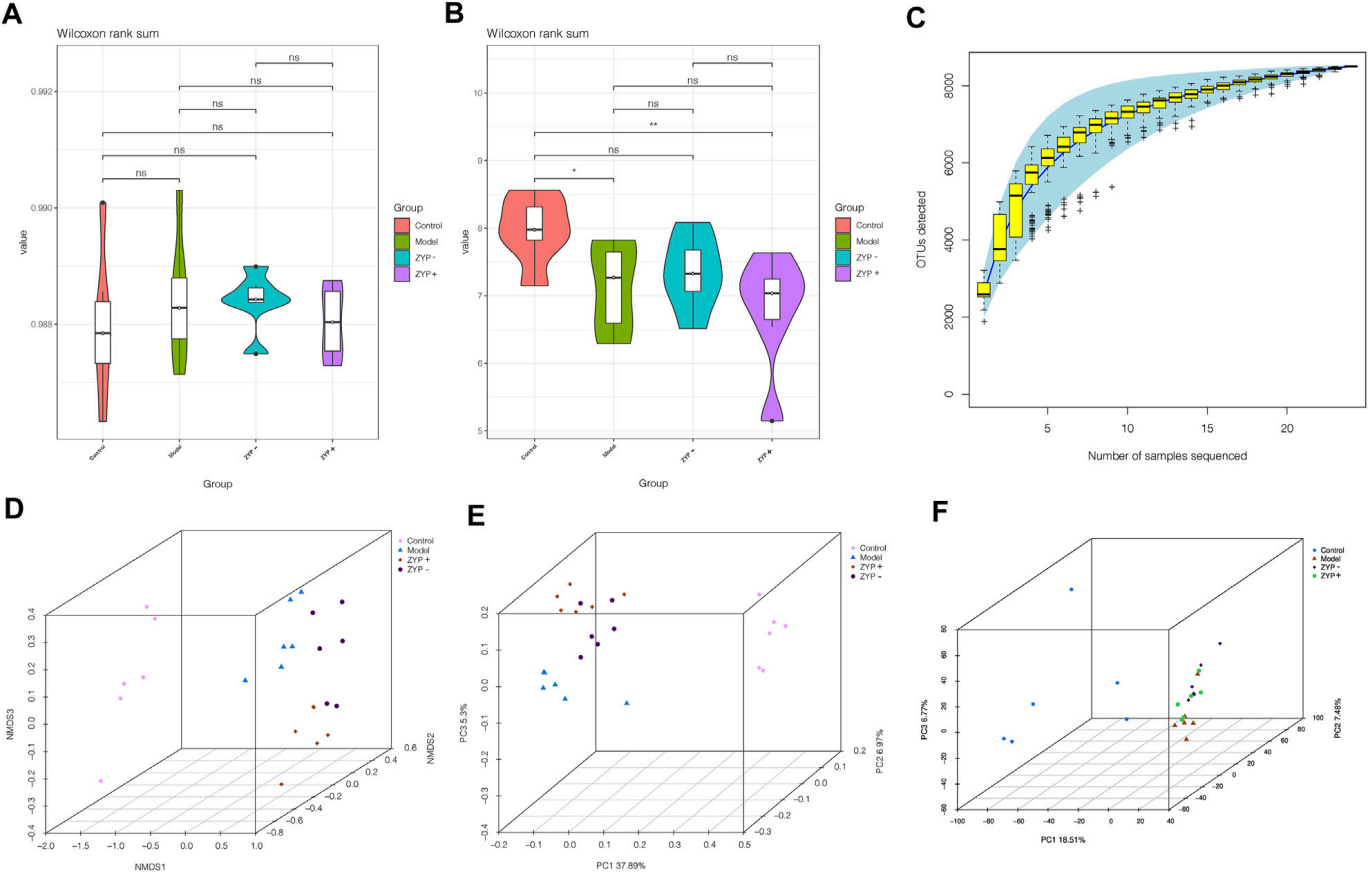
Alpha and beta diversity analysis of the OTUs. **(A)** Good diversity analysis; **(B)** Shannon diversity analysis; **(C)** Curve of SAC; **(D)** NMDS analysis; **(E)** PCoA analysis; **(F)** PCA analysis.

Alpha diversity is widely used for the analysis of microbial community diversity. Good’s Diversity Index, the Shannon Diversity Index, and the species accumulation curves (SAC) of each group are shown in Figures 6A–C. The violin plots of the Good’s diversity Index and the Shannon Diversity Index suggest that treatment with oral ZYP (low dose or high dose) had no significant effects on the alpha diversity of the fecal microbiota in cholestasis-induced rats. The amount of sequencing data was sufficient to reflect most microbial information in the samples.

Beta diversity analysis defines the extent of similarities and differences among microbial communities. The results of the Non-metric Multidimensional Scaling (NMDS), Principal Co-ordinates Analysis (PCoA, unweighted_unifrac), and PCA analyses are shown in Figures 6D–F. The data of the beta diversity analysis revealed distinct differences in Control, Model, ZYP−, and ZYP+ groups, illustrating that oral ZYP (either low or high doses) had a significant effect on the microbiota of cholestasis rats.

**FIGURE 7:**
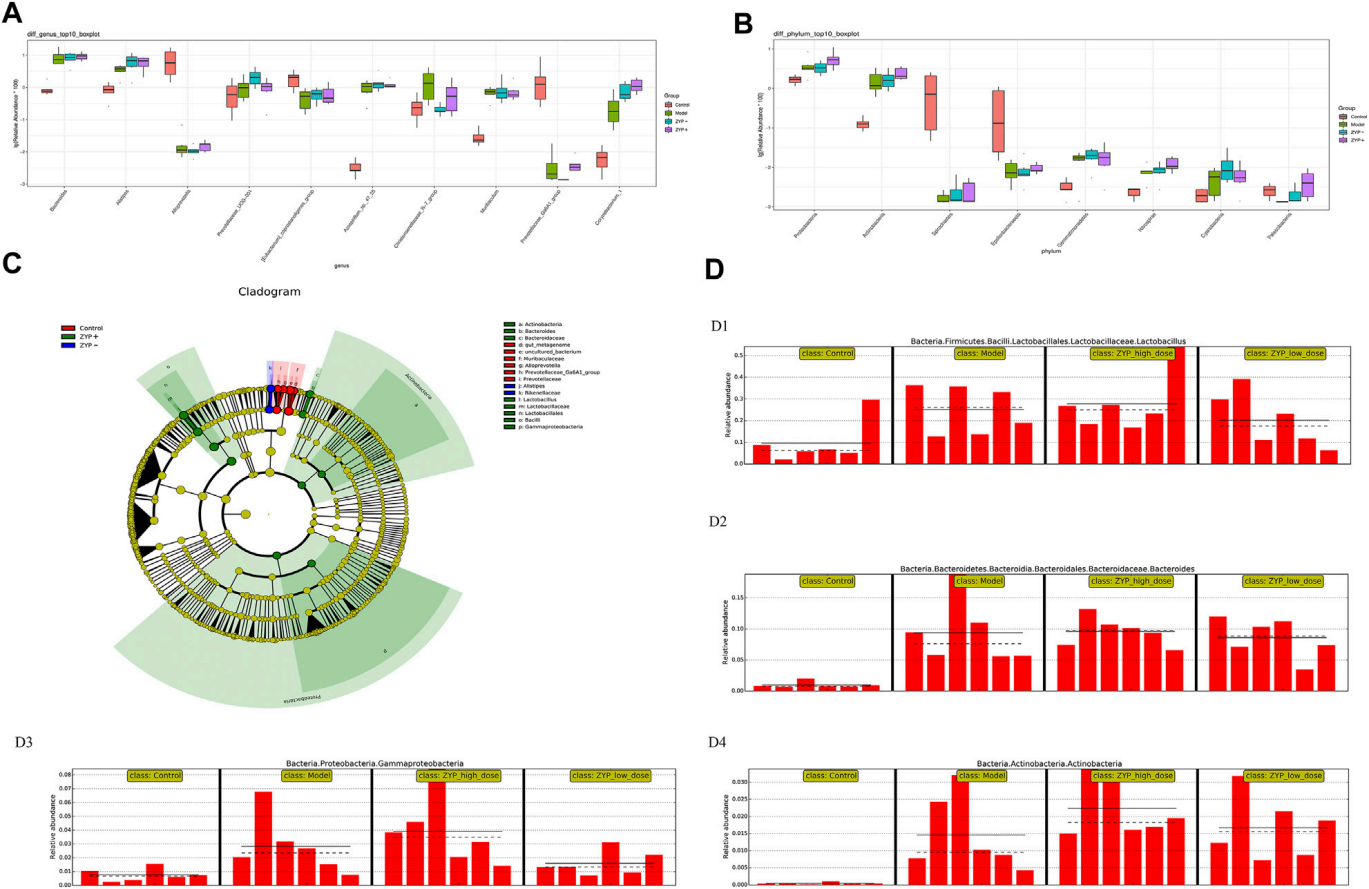
Statistical analysis of microbial multivariate. **(A)** One-way ANOVA analysis of genus level; **(B)** One-way ANOVA analysis of phylum level; **(C)** Example diagram of annotated branches of different species; **(D)** Histogram of relative abundance. D1, *Lactobacillus*; D2, *Bacteroides*; D3, *Gammaproteobacteria*; D4, *Actinobacteria*. The solid line is the mean value of the relative abundance and the dashed line is the median value of relative abundance.

To evaluate the differences in microbial diversity, one-way ANOVA was applied to identify significant alterations in the composition of the host fecal microbiota at the genus and the phylum levels during infection (Figures 7A,B), the results show that cholestasis and ZYP influenced species abundances. To filter which bacteria differed between the experimental groups, we conducted a LEfSe (linear discriminant analysis coupled with effect size measurements) analysis (Figure 7C). Overall, at the class and genus level, two classes and nine genera, specifically *Prevotellaceae_Ga6A1_group*, *uncultured_bacterium*, *Alloprevotella*, *gut_metagenome*, *Lactobacillus*, *Bacteroides*, *Gammaproteobacteria*, *Actinobacteria*, and *Alistipes*, were found to have significant differences. Of these, *Lactobacillus*, *Bacteroides*, *Gammaproteobacteria*, and *Actinobacteria* were the most representative bacteria in the ZYP+ treatment group. The above information is shown in Figure 7D.

### Potential Relationships Between Biochemical Indexes, Fecal Metabolites, and Fecal Microbiota

**FIGURE 8:**
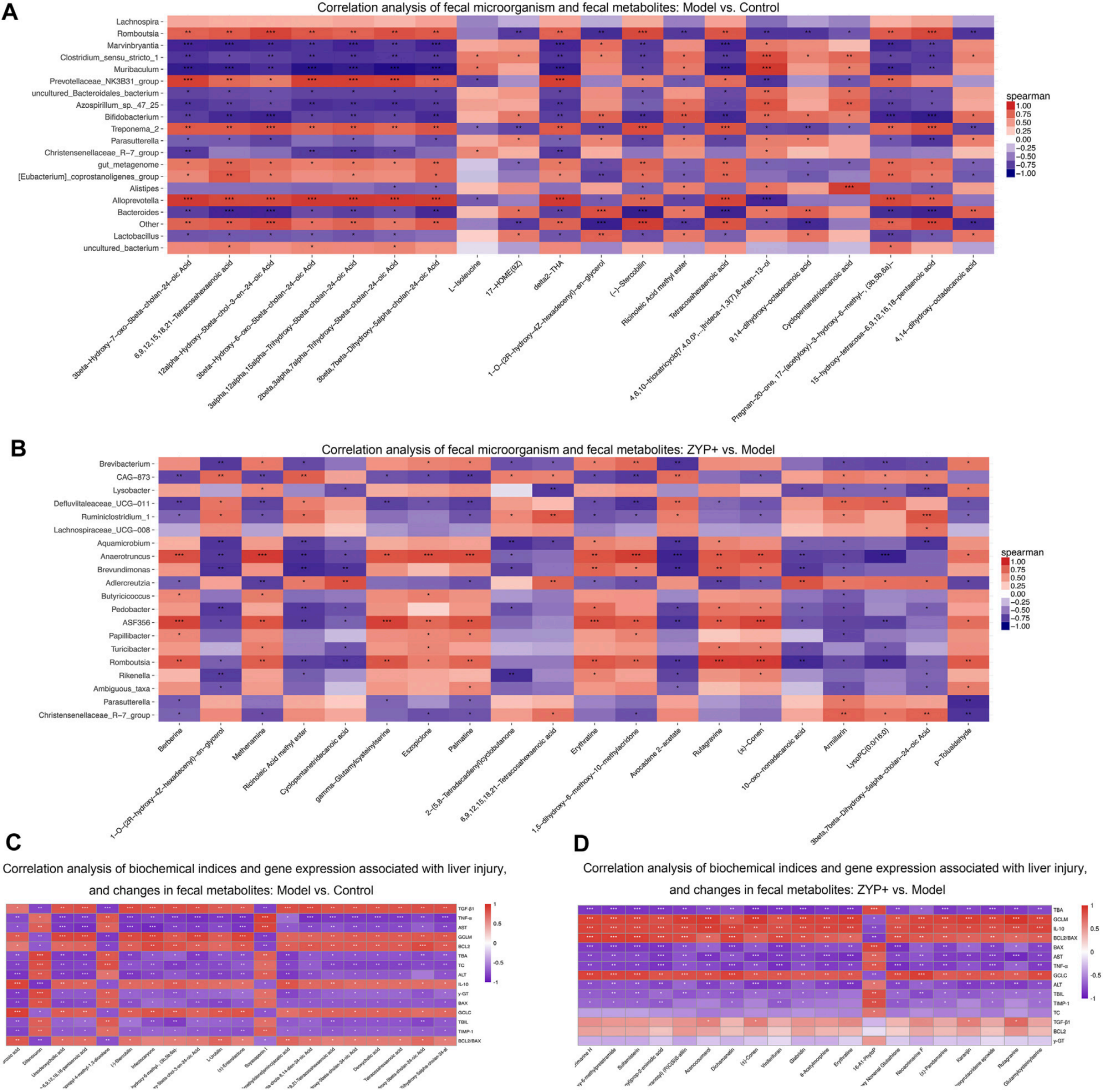
Combined analysis of blood biochemistry, gene expression changes, fecal microorganism composition, and fecal metabolism variables in liver injury. Correlation analysis of fecal microorganism and fecal metabolites **(A)** Cholestasis model vs Control; **(B)** ZYP+ vs Untreated Cholestasis model. Correlation analysis of biochemical indices and gene expression associated with liver injury, and changes in fecal metabolites **(C)** Cholestasis model vs Control; **(D)** ZYP+ vs Untreated Cholestasis model. *p < 0.05, **p < 0.01, ***p < 0.001. The correlation matrices were obtained from the cloud platform of Shanghai OE Biotech, Inc. (https://cloud.oebiotech.cn/task/).

To comprehensively analyze the relationships between biochemical indexes, fecal metabolites, and the genera of fecal microbiota, a correlation matrix was established calculating Spearman’s correlation coefficient. As shown in Figures 8A,B, the pairwise correlation between the Model vs Control and the ZYP+ vs Model groups showed consistently strong correlations between intestinal microorganism and fecal metabolites. Furthermore, six biochemical indices, (ALT, AST, TBIL, TBA, TC, γ-GT) and eight genes (TNF-α, TGF-β1, IL-10, TIMP-1, GCLC, GCLM, BCL2, and BAX) exhibited positive or negative correlations with a variety of metabolites (Figures 8C,D). These relationships suggested that the biochemical indices, gene expression associated with liver injury, fecal metabolites, and fecal microbiota can influence each other.

## Discussion

Cholestasis is a widespread clinical liver disease which requires new therapeutic options. In clinical practice, ZYP is an effective combination medicine for cholestasis treatment. The purpose of this study was to determine the therapeutic effects of ZYP on cholestasis, and to elucidate the related mechanisms by metabolomics and intestinal flora profiling.

Serum liver enzymes (including ALT, AST, and γ-GT), TC, TBA, and TBIL are important indices of the clinical manifestations of cholestatic hepatitis (Bouchier and Pennington, 1978; Gomez Selgas et al., 2014; Wu et al., 2021). TNF-α, IL-10, TIMP-1, GCLC, GCLM, BCL2, and BAX are common indicators used to evaluate the inflammatory response, oxidative stress, apoptosis, and fibrosis (Yamazaki, 1994; Golbar et al., 2017; Strasser and Vaux, 2018). The results of the present study suggested that ZYP, especially high-doses of ZYP (1.2 g/kg/d) could represent a potential anti-inflammatory, antioxidant, anti-fibrosis and anti-apoptosis effects. Furthermore, a clear improvement in liver damage was observed following ZYP treatment. Overall, these results indicated that ZYP could represent a potential therapeutic drug with protective effects against ANIT-induced cholestasis or following liver injury.

The diversity of fecal bacteria is associated with alterations and of the gut microbiome, and is often correlated with metabolism and immunity in patients with primary biliary cirrhosis (Lv et al., 2016; Wang et al., 2019). To explore the relevant fecal bacteria associated with ZYP treatment and cholestasis, we used 16S microbiome sequencing, which provides a measure of relative but not absolute bacterial abundance. In this study, we determined that *Lactobacillus*, *Bacteroides*, *Gammaproteobacteria*, and *Actinobacteria* were the most representative bacteria in the ZYP+ group (Figure 7). Thus, we hypothesized two possible explanations for the diversity observed in the Model and ZYP+ groups. First, most of the representative bacteria in the ZYP+ group, which may contribute to higher diversity, were beneficial bacteria, and were enhanced in the cholestasis Model group, such as *Lactobacillus*, *Bacteroidetes*, and *Actinobacteria*. Of these, bacteria of the *Lactobacillus* genus have been shown to increase resistance against environmental stresses, and are sensitive or weakly tolerant to acidic environments and bile acid.

*Bacteroidetes* and *Lactobacillus* express bile salt hydrolase (BSH) and are involved in the metabolism of bile (Han et al., 2017; Chen et al., 2020). *Actinobacteria* has been implicated in the modulation of gut permeability, the immune system, metabolism, and the gut-brain axis (Binda et al., 2018). Further, *Bacteroidetes* and *Lactobacillus* may be associated with elevated levels of bile acids, and the enhanced expression of *Actinobacteria* may be associated with immune-inflammatory and autoimmune response by inducing regulatory T-cells (O'Mahony et al., 2008; Lyons et al., 2010). High doses of ZYP enhance the expression of the above bacteria, which indicated an improvement in the expression of BSH or T cell activity; thus, the promotion of bile metabolism may partly explain the interventional mechanism of ZYP in cholestasis. Second, *Gammaproteobacteria*, a class of pathogenic bacteria, may exhibit detrimental potential and cause abnormal inflammatory activation (Williams et al., 2010), however, it also considered as survival tool for bacteria in the environment (Vazquez-Rosas-Landa et al., 2017). In this study, ZYP enhanced the expression of *Gammaproteobacteria*, which allows us to speculate that bacteria–bacteria interactions may play a role in the interventional mechanism of ZYP in cholestasis. However, the detailed mechanisms involved remain to be further explored.

Untargeted metabolomics may contribute to provide evidence that fecal microbiota metabolites infer host-microbiome co-metabolic effects (Griffiths et al., 2010), and may help further explain the potential mechanisms of ZYP in cholestasis, The metabolomes of fecal samples were analyzed *via* an untargeted LC-MS-based metabolomics approach. We identified 1090, 965, and 734 metabolites as differential metabolites between the Model vs Control, ZYP− vs Model, and ZYP+ vs Model group comparisons, respectively. To explore the underlying biological mechanisms and relationships involved in the differential production of these metabolites, we applied KEGG pathway analysis. As a result, eight pathways, namely bile secretion, steroid hormone biosynthesis, choline metabolism in cancer, pathways in cancer, histidine metabolism, prostate cancer, pancreatic cancer, and tryptophan metabolism were enriched when compared to metabolites generated by the Model vs Control and ZYP+ vs Model groups. Among these metabolic pathways, bile secretion plays a vital role in the health of an organism and blockage of bile secretion leads to cholestasis (Jia et al., 2020).

Recent studies have shown that steroid hormone biosynthesis involves the coordinate activity of several members of the cytochrome P450 superfamily of monooxygenases and is associated with fatty-acid degradation (Li et al., 2012; Zhao et al., 2019). Histidine metabolism and tryptophan metabolism also undergo amino acid metabolism. Previous studies have confirmed that amino acid metabolism has a clear correlation with cholestasis, as amino acids are the main precursors of glutathione biosynthesis (Yu et al., 2018; Li et al., 2019). Further, amino acid metabolism interferes with cholestasis by affecting glutathione synthesis and thus produces antioxidant stress, which is supported by other study showing that γ-GT catabolizes biliary glutathione and expands the pool of amino acid precursors required for conjugation (glycine directly and taurine through cysteine oxidation) (Chen MF. et al., 2019). However, in our study, correlation analysis between metabolomics and γ-GT showed that there was no significant correlation between the two in the ZYP+ vs model groups (Figure 8D); thus, the detailed mechanisms remain to be further explored. Altogether, the above findings suggest that perturbation of these metabolic pathways may act to improve metabolic activity, which ultimately explains the beneficial effects on cholestasis by ZYP intervention. In addition, the pathways in cancer, prostate cancer, pancreatic cancer, and choline metabolism in cancer were perturbed when comparing the Model vs Control groups and ZYP+ vs Model groups, which indicated that ZYP may also be promising as a potential anticancer agent.

To comprehensively analyze the relationships between biochemical indices, changes in the inflammatory response, apoptosis, and gene expression associated with fibrosis, fecal metabolites, and the impact on the genera of fecal microbiota, a correlation matrix was established using Spearman’s correlation coefficient analysis. Our findings confirmed a mutual effect between the biochemical indices, gene expression associated with liver injury, fecal metabolites, and fecal microbial genera, which suggested an underlying reciprocal relationship among these factors.

## Conclusion

In summary, the results of the present study demonstrated that ZYP exerts significant anti-cholestatic effects through a synergistic effect on fecal metabolism and fecal microbes. We also revealed that the altered fecal microbial composition achieved following ZYP treatment was marked by an increase of beneficial bacteria (*Bacteroidetes*, *Lactobacillus* and *Actinobacteria*) and pathogenic bacteria (*Gammaproteobacteria*) compared with the Model group, bacteria–bacteria interactions may play a role in the interventional mechanism of ZYP in cholestasis. In addition, oral administration of high-doses of ZYP also exerted a significant influence on fecal metabolomics against cholestasis in the rat, which might be associated with amino acid metabolism, steroid hormone biosynthesis, and bile secretion. These findings highlight that ZYP, an ancient classic formulation of Coptis-Evodia herb couple, may prevent cholestasis. Future research will improve the understanding of ZYP’s mechanisms and the pharmacological rationale underlying its therapeutic application.

## Data Availability

The datasets presented in this study can be found in online repositories. The names of the repository/repositories and accession number(s) can be found below: NCBI [accession: PRJNA720504]; Metabolights [accession: MTBLS2721].

## Abbreviation

CEHC, Coptis-Evodia herb couple; TCM, Traditional Chinese medicine; ZYP, Zhuyu Pill; ZYP-, ZYP-low dose; ZYP+, ZYP-high dose.
